# Study comparing the efficacy and renal safety for patients with diabetes switching from dapagliflozin to empagliflozin

**DOI:** 10.1007/s11096-020-01210-1

**Published:** 2020-11-30

**Authors:** Ai-Yu Yang, Hung-Chun Chen

**Affiliations:** 1 Department of Pharmacy, Kaohsiung Medical University Hospital, Kaohsiung Medical University, No.100, Ziyou 1st Rd., Sanmin Dist., Kaohsiung City, 807 Taiwan, Republic of China; 2 Division of Nephrology, Department of Internal Medicine, Kaohsiung Medical University Hospital, Kaohsiung Medical University, Kaohsiung, Taiwan, Republic of China; 3 Faculty of Medicine, College of Medicine, Kaohsiung Medical University, Kaohsiung, Taiwan, Republic of China

**Keywords:** Dapagliflozin, Empagliflozin, Defined daily dose, Sodium-glucose co-transporter-2 inhibitors

## Abstract

*Background* Previously, dapagliflozin was limited to patients with an estimated glomerular filtration rate (eGFR) ≥ 60 mL/min/1.73 m^2^, while empagliflozin can be used for those with an eGFR ≥ 45 mL/min/1.73 m^2^. Therefore dapagliflozin was switched to empagliflozin in many patients when eGFR decreased. However, the clinical efficacy and safety of these switcfhes are not clear. *Objective* In this study, we compared the efficacy and renal safety between patients switching from dapagliflozin to empagliflozin in patients. *Setting* This is a retrospective study of adult patients (aged ≥ 20 years) who had attended the Kaohsiung Medical University Hospital. *Method* This retrospective observational study included patients who were switched from dapagliflozin to empagliflozin. To assess the effect of other hypoglycemic drugs on efficacy, the types and dose alterations of other hypoglycemic drugs were classified on the defined daily dose (DDD). *Main outcome measure* The primary outcome measure was the change in hemoglobin A1c (HbA1c) level after 6 months. Patients with HbA1c levels at or lower than the baseline value after 6 months were defined as effective and patients with levels higher than the baseline were defined as invalid. Safety was evaluated by comparing the difference of eGFR between the baseline value and 6 months after treatment. *Result*s Overall, 111 patients were enrolled in the study. Six months after switching from dapagliflozin to empagliflozin, HbA1c significantly reduced, with no statistically significant difference observed in eGFR. In our study, 78 patients were assigned to the effective group (70.3%) and 33 patients were invalid (29.7%). When the other hypoglycemic drugs were grouped by total dosage, fasting plasma glucose and HbA1c only decreased significantly in the "DDD decrease" and "DDD increase" groups. *Conclusion *Our study showed that switching from dapagliflozin to empagliflozin in patients with type 2 diabetes was effective for blood glucose maintenance and caused no significant changes in renal function. In addition, compared to similar sodium-glucose co-transporter-2 inhibitors, other hypoglycemic drugs may be factors that influence the efficacy of sugar-lowering treatments.

## Impacts on practice

- The SGLT-2 inhibitors reduce glucose toxicity of proximal tubular cells; thus, they have a potential renal protective effect in patients with type 2 diabetes.
- Previously, dapagliflozin was limited to patients with an estimated glomerular filtration rate (eGFR) ≥ 60 mL/min/1.73 m2, while empagliflozin can be used for those with an eGFR ≥ 45 mL/min/1.73 m2.
- Therefore dapagliflozin was switched to empagliflozin in many patients when eGFR decreased. However, the clinical efficacy and safety of these switches are not clear.
- In this study, we compared the efficacy and renal safety between switching from dapagliflozin to empagliflozin in patients with type 2 diabetes.
- Our study showed that switching from dapagliflozin to empagliflozin in patients with type 2 diabetes was effective for blood glucose maintenance and caused no significant changes in renal function.

## Introduction

Diabetes is a chronic metabolic disease with a high prevalence. Approximately 40% of patients with diabetes develop chronic kidney disease [1]. Dapagliflozin (DPG) and empagliflozin (EPG) are sodium-glucose co-transporter-2 inhibitors (SGLT-2i) that reduce blood glucose levels primarily via the inhibition of SGLT-2 in the proximal tubules of the kidney and reduction of the reabsorption of glucose from the urine [2]. Ideal glycemic control can effectively reduce the risk of diabetic microvascular and macrovascular disease [1]. DPG and EPG were approved by the Food and Drug Administration (FDA) in January and August 2014, respectively. In June 2016, the FDA issued a drug warning and strengthened safety alerts on acute kidney damage for DPG [3].

Tang conducted a network meta-analysis of SGLT-2i and renal safety by collecting data on 511 patients with acute renal impairment/failure events from 53 randomized controlled trials. The results of the analysis showed that DPG increases the risk and EPG reduces the risk of these acute events [4]. However, large clinical trials that investigate renal adverse events as the endpoint have yielded differing results. In the DECLARE-TIMI 58 trial, secondary efficacy outcomes indicated that DPG treatment reduces overall kidney disease risk by 34% compared to the placebo [5]. The EMPA-REG trial indicated that the use of EPG (10 or 25 mg) reduces the risk of kidney disease development and progression by 39% compared to the placebo [6].

Previously, DPG was limited to patients with an estimated glomerular filtration rate (eGFR) ≥ 60 mL/min/1.73 m^2^, but EPG can be used in patients with an eGFR ≥ 45 mL/min/1.73 m^2^. The DERIVE study published by Fioretto in November 2018 demonstrated the efficacy and safety of DPG in patients with stage 3A chronic kidney disease (CKD) (CKD 3A: eGFR 45–59 mL/min/1.73 m^2^) [7]. In February 2019, the FDA approved the use of DPG in patients with an eGFR ≥ 45 mL/min/1.73 m^2^. Before the DERIVE study was published, patients prescribed DPG might have been switched to EPG due to decreased renal function. The similarity in clinical efficacy and effects on safety of this switch to a different SGLT-2i have not been demonstrated by relevant clinical studies.

## Aim of the study

In this study, we compared the efficacy and renal safety between switching from DPG to EPG in patients with type 2 diabetes (T2DM).

## Methods

### Patients

This is a retrospective study of adult patients (aged ≥ 20 years) who had attended the Kaohsiung Medical University Hospital (KMUH). KMUH is a medical center located in southern Taiwan with around 1600 beds and 6000 patient visits per day. The study population involved outpatients with T2DM and who had switched from DPG to EPG during the period from 1 January 2015 to 30 June 2017. This study is based on previously conducted studies and does not include any study with human participants or animals performed by any of the authors.

The inclusion criteria were as follows: (1) patients aged ≥ 20 years, eGFR ≥ 45 mL/min/1.73 m^2^, (2) those who had used DPG for 3 months, (3) continuous use of SGLT-2i, switched from DPG 10 mg to EPG 25 mg, (4) change to EPG and use for 6 months. Continuous was defined as the continuous use of SGLT-2i without a gap of ≧ 90 days, and switching was defined as the change from DPG to EPG. Switching over from DPG to EPG was at the discretion of the treating clinician.

During the study period, the patient's other hypoglycemic drugs and changes in their dosage were calculated using the "defined daily dose (DDD)" developed by the World Health Organization (WHO). Using metformin as an example, every 2 g is 1 DDD. In the case of compound medications, the WHO recommends that a fixed DDD be allocated based on the average use of different combinations, which is considered as the most appropriate.

The DDD of compound drugs defined in this study included: amaryl M (glimepiride 2 mg + metformin 500 mg) 1tab-1DDD, actosmet (pioglitazone 15 mg + metformin 850 mg) 1tab-1DDD, janumet (sitagliptin 50 mg + metformin 500 mg) 2tab-1DDD, galvusmet (vildagliptin 50 mg + metformin 850 mg) 2tab-1DDD.

**Fig. 1:**
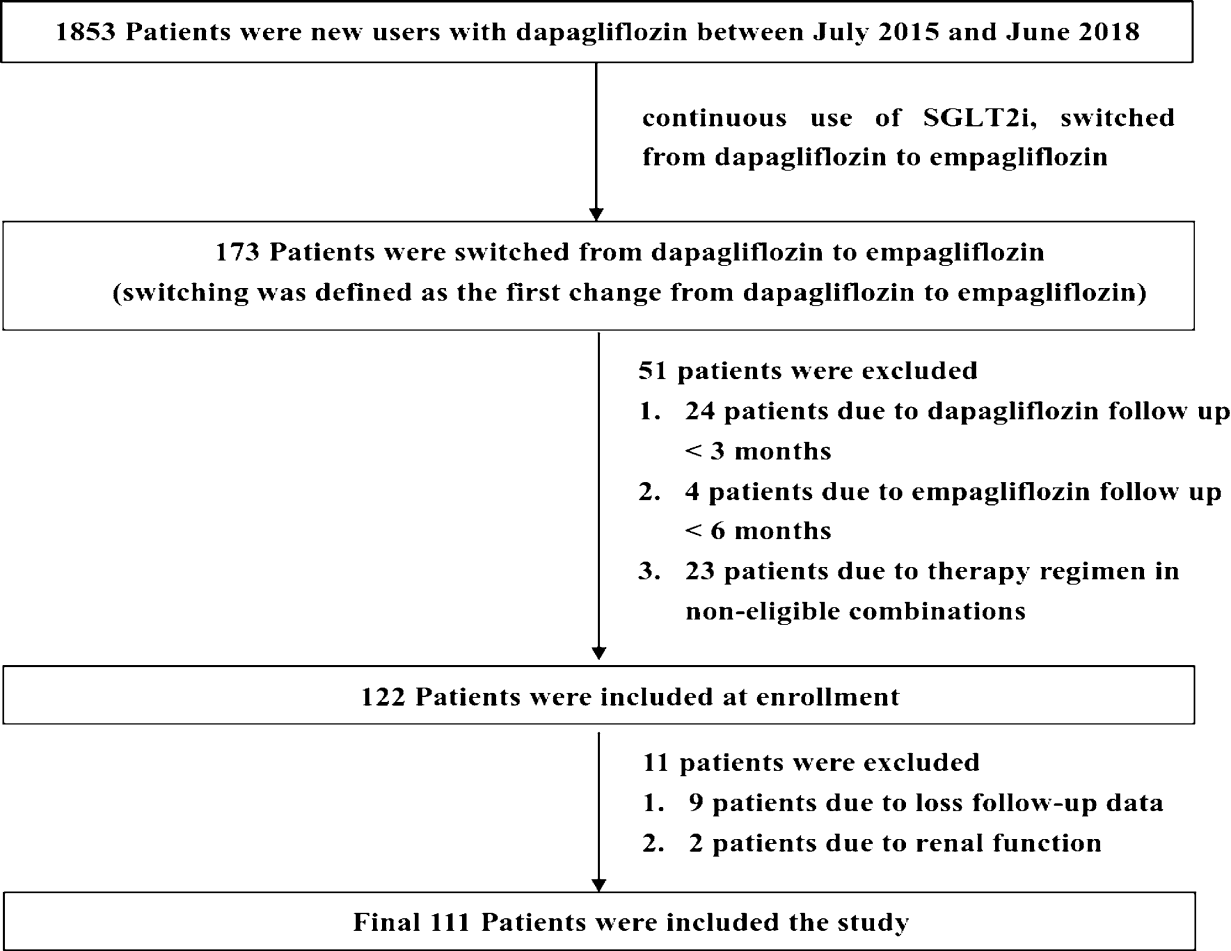
Flow chat of patient selection

The exclusion criteria were as follows: (1) missing HbA1c or eGFR level before or after treatment, (2) acute kidney failure (drastic changes in renal function) within 2 weeks before DPG treatment, (3) abnormal liver function before DPG treatment (serum alanine aminotransferase level 2 times higher than the normal level), (4) kidney transplant, (5) pregnancy or lactation, (6) concurrent anti-cancer or immunosuppressive treatment. A flow diagram of patient selection is shown in Fig. 1.

### Procedure

Patient data were collected using the electronic medical record system of the hospital, which included demographic data (age and sex), medical history (mainly focused on cardiovascular diseases), history of diabetes, and duration of follow-up.

Efficacy was evaluated as the difference between baseline HbA1c values and HbA1c values after 6 months of treatment. The types and dosages of other hypoglycemic drugs are also important factors that affect efficacy, so the DDD calculation was used for analysis. Cases were classified as "DDD decrease", "constant DDD ", and "DDD increase" based on the change in the total dosage of hypoglycemic drugs.

HbA1c reflects the status of blood glucose control in the body in the past 2–3 months, and efficacy was calculated as the difference between the baseline HbA1c value and HbA1c value after 6 months of treatment. The pre-treatment prescription was defined as the last prescription before the patient was switched to EPG. The post-treatment prescription was defined as the prescription at the 3- or 4-month follow-up.

We evaluated whether the hypoglycemic effect continued to be effective after drug switching. Patients with HbA1c levels at or below the baseline after 6 months were defined as the effective group and patients with levels above the baseline were defined as the invalid group. Additionally, the effects of changing the total dosage of other hypoglycemic drugs were compared using HbA1c values. Secondary outcomes included changes in fasting plasma glucose (FPG) and body weight (BW) after 6 months of treatment and were compared to the baseline values. Safety was evaluated as the difference between baseline eGFR values and eGFR values after 6 months of treatment. Patients using DPG for less than 3 months or EPG for less than 6 months were not included in the study or the evaluation of efficacy, and only the outpatient records of the prescribing physician were reviewed to record suspected adverse drug reactions.

The date on which the initial prescription of DPG was switched to EPG was designated as the index date, and the examination data in the 3 months before the index date was used as the baseline level. The study included cases where EPG was used continuously for over 6 months after switching from DPG, and the efficacy at 3 and 6 months was investigated. As glycated hemoglobin values reflect the status of blood glucose control in the body in the last 2–3 months, the data collection period for HbA1c at 6 months ranged from 6 to 9 months, and the ranges for the data collection period in the other months were analogous.

### Statistical analyses

The quantitative variables are expressed as median (interquartile range: 25–75%), and qualitative variables are expressed in percentage. The Chi-square test was used to compare the differences in gender, age, cardiovascular medical history between the effective and invalid group. For any value < 5, Fisher’s test was used. A Wilcoxon rank-sum test was used to compare the continuous changes between efficacy (HbA1c, BW, and eGFR) of the effective and invalid group. Logistic regression analysis was used to analyze the factors influencing the sustained and effective switching from DPG to EPG.

Kruskal Wallis test was used to compare the difference between the “DDD increase”, “constant DDD”, and “DDD decrease”. Friedman test was used to compare the intragroup changes in baseline values and after 3 and 6 months of treatment. Wilcoxon rank-sum test is applied to the comparison the intragroup changes in baseline values vs 3 vs 6 months of treatment. The results with a *p*-value of < 0.05 were considered statistically significant.

## Results

### Patient characteristics

Overall, 1853 patients were new DPG users between January 2015 and 30 June 2017. In total, 173 patients (9.34%) were switched from DPG to EPG between January 2015 and 30 June 2017. The original number of patients after the screening was 122 (flow diagram is presented in Fig. 1). Two cases lacked follow-up records during the admission period, with nine cases demonstrating a lack of blood test values during treatment. Finally, 111 patients were enrolled in the study. Based on changes in the total dosage of hypoglycemic drugs, the cases were classified as "DDD decrease", "constant DDD", and "DDD increase". General data, history of cardiovascular-related diseases, and changes of clinical parameters are presented in Table 1.

**Table 1:** Comparison of clinical characteristics and changes of clinical parameters between the types and dose alterations of other hypoglycemic drugs

	Overall patients (N = 111)	DDD decrease (N = 24)	Constant DDD (N = 64)	DDD increase (N = 23)	*P*-value in DDD groups^#^
Men	67 (60.4%)	15 (62.5%)	37 (57.8%)	15 (65.2%)	0.800
Age, years	64.00 (57.00–70.50)	69.00 (57.50–76.50)	62.00 (57.00–67.75)	67.00 (53.00–71.00)	0.065
*Co morbidities*					
Hypertension	78 (70.3%)	16 (66.7%)	43 (67.2%)	19 (82.6%)	0.347
Dyslipidemia	104 (93.7%)	23 (95.8%)	60 (93.8%)	21 (91.3%)	0.815
Cardiovascular disease	31 (27.9%)	7 (29.2%)	15 (23.4%)	9 (39.1%)	0.351
*Concomitant medication*					
Metformin	48 (43.2%)	7 (29.2%)	30 (46.9%)	11 (47.8%)	0.290
Sulfonylurea	75 (67.6%)	17 (70.8%)	41 (64.1%)	17 (73.9%)	0.638
Pioglitazone	40 (36.0%)	8 (33.3%)	22 (34.4%)	10 (43.5%)	0.703
DDP -4 inhibitor	22 (19.8%)	2 (8.3%)	17 (26.6%)	3 (13.0%)	0.106
Acarbose	27 (24.3%)	6 (25.0%)	16 (25.0%)	5 (21.7%)	0.949
Insulin	40 (36.0%)	14 (58.3%)	17 (26.6%)	9 (39.1%)	0.021*
GLP-1 agonist	2 (1.8%)	2 (8.3%)	0	0	0.025*
*Fasting plasma glucose(FPG), mg/dL*					
Baseline	149.00 (122.50–170.25)	151.00 (117.00–168.00)	141.50 (118.75–167.75)	153.00 (139.00–177.00)	0.427
3 months	132.00 (109.50–154.00)	116.00 (98.50–145.00)	138.50 (114.00–156.50)	115.00 (108.00–139.00)	0.041*
6 months	129.50 (110.00–154.25)	116.00 (97.00–132.00)	137.00 (114.00–162.50)	118.00 (110. 00–142.00)	0.049*
*p* value of FPG in the Intragroup^##^	< 0.001*	0.016*	0.237	0.001*	
*Hemoglobin A1c(HbA1c), %*					
Baseline	7.70 (7.10–8.60)	8.00 (7.13–8.78)	7.60 (7.00–8.58)	7.90 (7.20–8.50)	0.527
3 months	7.60 (6.90–8.20)	7.65 (6.85–7.98)	7.50 (6.83–8.73)	7.60 (7.10–8.00)	0.861
6 months	7.50 (6.90–8.30)	7.60 (6.73–8.15)	7.45 (6.90–8.48)	7.50 (6.90–8.00)	0.845
*p* value of HbA1c in the Intragroup^##^	< 0.001*	0.025*	0.301	0.010*	
*eGFR, mL/ min/ 1.73 m*^2^					
Baseline	80.00 (63.00–96.00)	78.50 (61.50–89.50)	81.00 (65.25–99.00)	78.00 (58.00–108.00)	0.766
3 months	78.00 (66.00–95.00)	76.00 (63.00–91.25)	78.50 (66.25–96.00)	76.00 (58.00–101.00)	0.728
6 months	79.00 (61.00–95.00)	76.50 (60.00–90.50)	79.50 (64.50–95.75)	79.00 (54.00–98.00)	0.747
*p* value of eGFR in the Intragroup^##^	0.235	0.220	0.910	0.091	
*Body weight(BW), kg*					
Baseline	70.40 (62.80–83.60)	69.80 (58.60–81.40)	71.45 (65.70–88.80)	68.90 (62.60–80.20)	0.325
3 months	70.60 (62.80- 83.20)	69.80 (57.70- 80.30)	71.75 (64.63- 88.88)	70.10 (63.90- 80.45)	0.408
6 months	70.40 (63.40–82.00)	69.20 (56.90–80.60)	71.00 (65.25–88.60)	70.80 (63.55–81.30)	0.378
*p* value of BW in the Intragroup^##^	0.017*	0.060	0.060	0.481	
*DDD levels*					
Before switch	2.33 (1.67–3.25)	2.67 (1.95–3.33)	2.33 (1.35–3.25)	2.25 (1.72–3.00)	0.551
After switch	2.25 (1.33–3.25)	1.78 (0.97–2.91)	2.33 (1.35–3.25)	2.50 (1.95–3.50)	0.114
*p* value of DDD levels in the Intragroup^##^	0.154	< 0.001*	1.000	< 0.001*	

Non-parametric data presented in median (interquartile range: 25–75%)

*DDD* defined daily dose (the types and dose alterations of other hypoglycemic drugs were classified as increased, unchanged, or decreased); DDP -4, dipeptidyl peptidase-4; eGFR, estimated glomerular filtration rate (mL/min/1.73 m^2^); GLP-1, glucagon-like peptide-1

^#^Kruskal Wallis test was used to compare the difference between the “DDD increase”, “constant DDD”, and “DDD decrease”

^##^Friedman test was used to compare the intragroup changes in baseline values and after 3 and 6 months of treatment

^*^p-value < 0.05 were considered statistically significant

In total patients, HbA1c and FPG decreased significantly six months after switching from DPG to EPG (*p* < 0.001). BW showed statistically significant difference (*p* = 0.017) and no statistically significant difference was observed in eGFR (*p* = 0.235). No significant differences were observed in FPG, HbA1c, BW, eGFR, and DDD levels before drug switching in the between DDD related three groups. Six months after switching from DPG to EPG, decreased FPG and HbA1c values only in the "DDD decrease" and "DDD increase" groups were statistically significant.

### Efficacy and renal safety

Table 2 shows the comparison of FPG, HbA1c, eGFR, BW levels between 3 and 6-month values and the baseline values in the effective (78 patients, 70.3%) and invalid group (33 patients, 29.7%). There was no difference in clinical characteristics (comorbidities and concomitant medication) between the two groups. In baseline values, there was a significant difference in the HbA1c levels between the two groups before treatment, effective group (median: 7.9%) showed a high level than invalid group (median: 7.5%). However no difference was observed in the FPG, BW, and eGFR level between the two groups. Patients in both the groups (effective and invalid group) showed statistically significant reduction in the HbA1c levels after 6 months. After 6 months, 30 patients (27.0%) (all in effective group) achieved the diabetes treatment goal (HbA1c < 7%).

**Table 2:** Comparison of clinical characteristics and changes of clinical parameters between the effective and invalid group in overall patients

	Effective (N = 78)	Invalid (N = 33)	*P*-value^#^
Men	49 (62.8%)	18 (54.5%)	0.415
Age, years	65.00 (57.00–70.00)	62.00 (53.50–70.50)	0.677
*Co morbidities*			
Hypertension	58 (74.4%)	20 (60.6%)	0.147
Dyslipidemia	73 (93.6%)	31 (93.9%)	0.945
Cardiovascular disease	20 (25.6%)	11 (33.3%)	0.409
*Concomitant medication*			
Metformin	32 (41.0%)	16 (48.5%)	0.468
Sulfonylurea	54 (69.2%)	21 (63.6%)	0.565
Pioglitazone	32 (41.0%)	8 (24.2%)	0.092
DPP -4 inhibitor	14 (17.9%)	8 (24.2%)	0.447
Acarbose	20 (25.6%)	7 (21.2%)	0.619
Insulin	28 (35.9%)	12 (36.4%)	0.963
GLP-1 agonist	0	2 (2.6%)	1.000
*FPG, mg/dL*			
Baseline	142.00 (123.25–166.75)	152.50 (118.50–173.50)	0.530
3 months	116.00 (107.00–144.00)	149.00 (126.25–159.75)	0.002*
6 months	123.50 (106.00–145.75)	138.50 (122.50–165.00)	0.006*
*p* value of FPG in the Intragroup^##^	< 0.001*	0.990	
*Hemoglobin A1c(HbA1c), %*			
Baseline	7.90 (7.10–8.73)	7.50 (6.80–8.00)	0.040*
3 months	7.50 (6.68–8.13)	7.70 (7.10–8.45)	0.108
6 months	7.40 (6.68–8.10)	8.00 (7.40–8.60)	0.004*
*p* value of HbA1c in the Intragroup^##^	< 0.001*	< 0.001*	
*eGFR, mL/min/1.73 m*^*2*^			
Baseline	78.00 (58.75–95.50)	81.00 (70.00–97.00)	0.331
3 months	76.00 (64.75–92.75)	83.00 (67.50–98.50)	0.253
6 months	76.50 (59.00–95.00)	84.00 (65.50–102.00)	0.120
*p* value of eGFR in the Intragroup^##^	0.036*	0.586	
*Body weight(BW), kg*			
Baseline	70.20 (62.70–83.50)	73.50 (63.30–85.40)	0.961
3 months	70.50 (62.80–83.25)	73.00 (63.40–85.30)	0.841
6 months	70.20 (63.70–82.05)	72.90 (62.80–85.00)	0.831
*p* value of BW in the Intragroup^##^	0.074	0.149	
*DDD levels*			
Before switch	2.33 (1.63–3.31)	2.25 (1.70–3.08)	0.809
After switch	2.32 (1.48–3.37)	2.20 (1.25–3.00)	0.476
*p* value in the Intragroup^##^	0.511	0.527	

Non-parametric data presented in median (interquartile range: 25–75%)

^#^Wilcoxon rank-sum test was used to compare the effective and invalid group

^##^Friedman test was used to compare the intragroup changes in baseline values and after 3 and 6 months of treatment

^*^p-value < 0.05 were considered statistically significant

DPP -4, dipeptidyl peptidase-4; eGFR, estimated glomerular filtration rate (mL/min/1.73 m^2^); *FPG* fasting plasma glucose; GLP-1, glucagon-like peptide-1

The effective group displayed HbA1c decreased significantly, whereas the invalid group displayed HbA1c increased significantly. In this study, multivariate logistic regression (adjusted) was used to analyze the influencing factors that may have affected the effective group and these were shown to be related to baseline HbA1c in Table 3.

**Table 3:** Univariate and multivariate logistic regression models to evaluate the association between predictors and achieving target in effective group

	Univariate model		Multivariable model	
	β	*P*	β	*P*
Gender, female	0.990	0.988		
Age, years	1.011	0.744		
Hypertension	0.429	0.216		
Dyslipidemia	0.726	0.828		
Cardiovascular disease	2.205	0.421		
Metformin	0.999	0.998		
Sulfonylurea	2.024	0.356		
Pioglitazone	0.661	0.612		
DPP -4 inhibitor	0.681	0.620		
Acarbose	1.447	0.635		
Insulin	2.601	0.221		
GLP-1 agonist	0.001	1.000		
DDD staying the same	0.910	0.939		
DDD increase	0.620	0.578		
Baseline FPG	0.984	0.119		
Baseline HbA1c, %	5.058	0.005	1.486	0.043*
Baseline eGFR	0.998	0.882		
Baseline BW	0.992	0.762		

*BW* Body weight; *DDD* defined daily dose; DPP -4, dipeptidyl peptidase-4; *eGFR* estimated Glomerular filtration rate (mL/min/1.73 m^2^); *FPG* Fasting plasma glucose; GLP-1, glucagon-like peptide-1; HbA1c, hemoglobin A1c

^*^p-value < 0.05 were considered statistically significant

### Adverse events

In our study, 173 patients were switched from DPG to EPG; DPG (*n* = 24) follow up < 3 months and EPG (*n* = 4) follow up < 6 months. Uncontinuous medications or changing medications may be related to adverse reactions. In our study, if uncontinuous medications or changing medications, the reason was determined. Outpatient records during the medication period were examined for possible adverse reactions.

Before drug switching (DPG follow up < 3 months), 10 cases (41.7%) demonstrated eGFR < 60 mL/min/1.73 m^2^, two presented unstable blood glucose control, one patient reported a headache, and one patient reported gastrointestinal discomfort; other relevant records were missing. After drug switching (EPG follow up < 6 months), two patients were lost to follow-up, and two patients were switched to other types of hypoglycemic drugs due to intolerance of urinary tract symptoms.

## Discussion

According to International Diabetes Federation statistics, half of diabetes patients worldwide are from Asian countries [8]. The ADVANCE study (Action in Diabetes and Vascular Disease study) conducted by Clarke et al. indicated that the incidence of renal complications in Asian diabetic patients is significantly higher than that in Eastern European patients [9]. A study by Satirapoj et al. indicated that SGLT-2i reduce glucose toxicity of proximal tubular cells; thus, they have a potential renal protective effect in patients with T2DM [10]. As Asian patients have a high risk of diabetic nephropathy, the use of SGLT-2i in Asian patients plays an important role.

### Efficacy and renal safety

Bell et al. conducted a retrospective study to investigate whether switching from canagliflozin to EPG would worsen blood glucose control. They discovered that 3 months after switching drug treatments, HbA1c level rises by a statistically significant amount (57.7 ± 11.2 to 60.1 ± 13.2 mmol/mol, *p* = 0.028); however, there are no differences in BW, blood pressure, and blood lipids [11]. Previously, there have been no studies related to switching from DPG to EPG. Our study showed that 6 months after switching to EPG, FPG and HbA1c decreased significantly, with no significant difference in eGFR. The results that switching patients with T2DM from DPG to EPG was effective for blood glucose maintenance and caused no significant changes in renal function.

The type and dosage of other hypoglycemic drugs may be important variables affecting efficacy. However, hypoglycemic drug types are complicated, and their hypoglycemic efficacy varies widely. Therefore, the cases were divided into groups using the DDD developed by the WHO for evaluation. In the case of efficacy, a significant decrease was observed in FPG and HbA1c only in the "DDD decrease" and "DDD increase" groups. Shyangdan et al. used a network meta-analysis to perform indirect comparisons of the HbA1c-reducing effect of EPG 25 mg (0.64%) and DPG 10 mg (0.54%). When the two drug treatments are administered together, the efficacy of the two SGLT-2i do not exhibit a significant difference [12].

Compared to the results of this study, when other hypoglycemic drugs were not switched (defined as the same total DDD, DDD staying the same group), the difference in the effect of switching from DPG to EPG did not attain significance. Although no significant differences were observed in DDD levels before drug switching in the between DDD related three groups. But the more users with insulin in "DDD decrease" and "DDD increase" groups. Compared to similar SGLT-2i, other hypoglycemic drugs may be factors affecting the efficacy of glucose-lowering treatments.

In this study, logistic regression analysis was used to analyze the influencing factors that may have affected the effective group and these were shown to be related to baseline HbA1c. Hong et al. conducted a retrospective study to analyze the clinical characteristics of good responders (defined as HbA1c < 7.0% or a decrease in HbA1c of more than 1.0% after 12 weeks of treatment). The group with high initial blood glucose values (HbA1c and FPG) and not under insulin therapy that responded well to other blood-glucose-lowering drugs or increased SGLT-2i [13].

Clinical studies of SGLT-2i have shown that eGFR decreases in a dose-dependent manner within weeks after initial use [6, 7, 14, 15]. In the present study, no difference was observed in eGFR with 6 months of continuous EPG use, which may be related to the fact that all patients admitted to the study had been using DPG for 3 months.

### Adverse events

A meta-analysis of randomised controlled trials (RCT) by Donnan et al. showed that when compared with placebo. Urinary tract infections (UTI) were the most frequently reported adverse events. Related to SGLT-2i, we found no significant in UTI risk (RR 1.02; 95% CI 0.95 to 1.09). Only DPG increases the risk of UTIs (RR 1.21; 95% CI 1.02 to 1.43) [16]. Another meta-analysis by Puckrin et al. showed SGLT-2i increased similar risk of genital tract infection versus placebo: RR 3.45 (95% CI 2.55–4.66) for DPG, and RR 3.11 (95% CI 2.29–4.21) for EPG [17].

In our study, SGLT-2i were switched in 173 patients. In the case of SGLT-2i switching (use of DPG < 3 months), the primary reason may be associated with renal function. After SGLT-2i switching (use of EPG < 6 months), it may be associated with adverse drug reactions and patient intolerance.

When the medical history was ascertained through outpatient medical records to investigate possible reasons for SGLT-2i switching in the included patients, decreased renal function was noted in only 5 cases, and symptoms of persistent UTI or vaginal pruritus were documented in 3 cases each. Furthermore, other adverse reactions, including polyuria, pruritus, rash, headache, and hunger, were reported in 1–2 cases each. Additionally, outpatient medical records indicated preventing the risk of SGLT-2i in urinary tract or genital infections. When an outpatient prescription is issued, health education should be enhanced (water supplementation, no urination, and good hygiene habits).

## Limitations

This study was a retrospective analysis of medical records and it was not possible to obtain records of the diets and living habits of the patients. Although patient test values were monitored and adverse events were recorded, minor adverse reactions may have been neglected depending on individual differences and tolerance to symptoms. Secondly, although differences in treatment due to drug dose were considered in this study, the research subjects were outpatients and compliance with medication might have affected the evaluation of efficacy and safety. Furthermore, as this study was limited to a single center, the number of cases enrolled was limited and the follow-up period was only 6 months. Long-term, large-scale clinical studies are needed to draw reliable conclusions on the effect of switching between different SGLT-2i on efficacy and safety.

## Conclusions

Our study showed that switching patients with T2DM from DPG to EPG was effective for blood glucose maintenance and caused no significant changes in renal function. In the present study, after switching to EPG treatment, decreased HbA1c was the only significant difference among the groups, with the "DDD decrease" and "DDD increase" groups. This suggests that compared to similar SGLT-2i, other hypoglycemic drugs may be factors influencing the efficacy of glucose-lowering treatments.

## Data Availability

Data sharing is not applicable to this article as no new data were created or analysed in this study.
